# Impact of Dietary Niacin on Metabolic Dysfunction-Associated Steatotic Liver Disease in Mediterranean Subjects: A Population-Based Study

**DOI:** 10.3390/nu16234178

**Published:** 2024-11-30

**Authors:** Maria Antentas, Marina Idalia Rojo-López, Pau Vendrell, Minerva Granado-Casas, Idoia Genua, Berta Fernandez-Camins, Joana Rossell, Julia Niño-Narvión, Estefanía Moreira, Esmeralda Castelblanco, Emilio Ortega, Bogdan Vlacho, Nuria Alonso, Didac Mauricio, Josep Julve

**Affiliations:** 1 Institut de Recerca Sant Pau (IR SANT PAU), Sant Quintí 77-79, 08041 Barcelona, Spain; maantentas@gmail.com (M.A.); nut.marina.rojo.l@gmail.com (M.I.R.-L.); igenua@santpau.cat (I.G.); fernandezcamins@gmail.com (B.F.-C.); joanarossell@gmail.com (J.R.); aemoreira@gmail.com (E.M.); bvlacho@santpau.cat (B.V.); didacmauricio@gmail.com (D.M.); 2 Grup de Diabetis d’Atenció Primària (DAP-Cat), Unitat de Suport a la Recerca Barcelona, Fundació Institut Universitari per a la Recerca a l’Atenció Primària de Salut Jordi Gol i Gurina, 08007 Barcelona, Spain; pauvendrellt@gmail.com (P.V.); minervagranado@outlook.com (M.G.-C.); 3 Department of Nursing and Physiotherapy, University of Lleida, 25198 Lleida, Spain; 4 Research Group of Health Care (GReCS), IRBLleida, 25198 Lleida, Spain; 5 Centro de Investigación Biomédica en Red de Diabetes y Enfermedades Metabólicas Asociadas (CI-BERDEM), Instituto de Salud Carlos III, 28029 Madrid, Spain; 6 Department of Endocrinology and Nutrition, Hospital de la Santa Creu i Sant Pau, 08041 Barcelona, Spain; 7 Division of Endocrinology, Metabolism and Lipid Research, Department of Internal Medicine, Washington University School of Medicine, St. Louis, MO 63110, USA; esmeraldacas@gmail.com; 8 Department of Medicine, Universitat Autònoma de Barcelona, 08193 Bellaterra, Spain; eortega1@clinic.cat; 9 Department of Endocrinology and Nutrition, Institut d’Investigacions Biomèdiques August Pi i Sunyer, Hospital Clínic, 08036 Barcelona, Spain; 10 Centro de Investigación Biomédica en Red de Fisiopatología de la Obesidad y Nutrición (CIBEROBN), Instituto de Salud Carlos III, 28029 Madrid, Spain; 11 Department of Endocrinology and Nutrition, Hospital de la Germans Trias i Pujol, 08916 Barcelona, Spain; nalonso32416@yahoo.es; 12 Department of Medicine, University of Vic—Central University of Catalonia, 08500 Vic, Spain

**Keywords:** hepatic steatosis, vitamin B3, niacin, tryptophan, fatty liver, case-control study

## Abstract

Background: The impact of dietary niacin on metabolic dysfunction-associated steatotic liver disease (MASLD) is elusive. This sub-study aimed to investigate the relationship between dietary niacin intake and the presence of MASLD in participants from two Catalonian cohorts. Methods: A total of 222 subjects with MASLD were age- and sex-matched to 222 non-MASLD subjects. Dietary nutrients were analyzed using a validated food frequency questionnaire (FFQ). Dietary niacin and other nutrients were adjusted for total energy intake. MASLD was defined by a Fatty Liver Index (FLI) of >60 and by having at least one component of metabolic syndrome. The association between niacin intake (distributed into tertiles) and the presence of MASLD was assessed using multivariate logistic regression. Potential non-linear relationships were also analyzed through restricted cubic spline regression (RCS). Results: Our data revealed that subjects with MASLD had worse metabolic profiles. The dietary intake of niacin did not differ between subjects with and without MASLD. Even after adjusting for different confounding variables, i.e., sociodemographic variables, smoking status, physical activity, and cardiometabolic comorbidities, no significant associations were observed between higher intakes of niacin (tertiles 2 and 3) and the presence of MASLD: odds ratio (95% confidence) second tertile: 0.99 (0.89–1.09); third tertile: 0.98 (0.89–1.10). However, RCS analysis uncovered a significant non-linear dose-response association between dietary niacin intake and odds of MASLD. Specifically, such analysis revealed that MASLD risk was decreased in subjects with niacin intake values of <35 mg/day. Conclusions: Our data showed that dietary niacin intake was associated with lower odds of MASLD in a Mediterranean population; however, our logistic regression analysis failed to reveal significant associations between the intake of niacin and the risk of MASLD. Further research is warranted to establish a causal relationship between dietary niacin interventions and MASLD.

## 1. Introduction

Metabolic dysfunction-associated steatotic liver disease (MASLD) is a dysmetabolic condition of the liver that is frequently linked to excessive caloric intake, overweight/obesity, or impaired insulin signaling in the absence of excessive alcohol consumption, autoimmune factors, infection, and/or other liver disorders [1,2]. Clinically, MASLD is defined by the presence of excessive triglyceride accumulation in hepatocytes (hepatic steatosis) and the presence of at least one cardiometabolic risk factor, i.e., type 2 diabetes mellitus (T2DM), obesity, or one of the components of metabolic syndrome [3,4]. Epidemiologically, its prevalence is steadily increasing worldwide. MASLD affects about one-third of the general population, reaching values closer to 55% in subjects with T2DM [1,5].

MASLD can be a reversible condition [6]. With emerging pharmacological therapies to specifically prevent or regress MASLD still under investigation, current clinical management consists of lifestyle modifications, including healthy diet/nutritional counseling and physical activity recommendations [1,2]. In particular, caloric restriction, limiting the energy intake to 500 to 750 kcal/day in combination with adherence to Mediterranean Diet (MetDiet) patterns, is currently considered the recommended dietary pattern in the treatment of MASLD [6]. In MetDiet, about 35% of calories come from low-glycemic carbohydrates, 45% from monounsaturated fatty acids, and 15–20% from proteins [6,7]. The favorable effects of MetDiet components on hepatic fat accumulation have been assessed in several studies, particularly focusing on the relative proportions of dietary carbohydrates and fats [8,9,10]. In contrast, the contribution of protein intake, both quantitatively and qualitatively, to MASLD remains poorly studied [11].

Niacin, also known as vitamin B3, encompasses both nicotinamide and nicotinic acid, two forms of the vitamin that contribute to essential metabolic processes in the body. Importantly, tryptophan, an essential amino acid from dietary protein, also contributes to the niacin pool in an organism [12]. Thus, the main dietary sources of niacin are predominantly protein-based, such as beef, poultry, eggs, fish, dairy, legumes, some cereals, and yeast, which are also high in tryptophan [13]. In this respect, the term “niacin equivalents” (NE) is used to estimate niacin intakes and requirements from other macronutrients, particularly proteins, in humans, where 1 mg NE corresponds to 60 mg tryptophan [12,13,14]. By meeting the daily protein needs of adults, typically achieved by consuming a total of 100 g of protein per day, the daily niacin requirements can by far be fulfilled [13].

Compelling evidence suggests that low levels of the oxidative form of nicotinamide adenine dinucleotide (NAD+) are a common feature of MASLD [15,16,17,18,19,20]. In this regard, the use of NAD+ precursors, i.e., niacin derivatives and tryptophan, has been assessed as a NAD+-increasing approach to ameliorate experimental MASLD in preclinical models [21,22,23,24,25,26,27,28]. Additionally, niacin may also inhibit hepatic lipogenesis via GPR109A-mediated signaling pathway [29]. Despite this, data on the potential effect of NAD+ precursor-based interventions on MASLD in humans are limited and inconsistent [2,30,31]. A few studies have suggested that dietary niacin may help ameliorate MASLD [32,33]. Supporting this, a two-year non-randomized intervention study showed that higher niacin intake reduced liver fat content in 58 subjects with MASLD, of whom 23 reversed their disease [33]. In contrast, a placebo-controlled clinical trial of nicotinamide riboside supplementation (250 mg/day) and pterostilbene (50 mg/day) for 6 months only showed a reduction in signs of liver inflammation but not steatosis in humans with MASLD/MASH [31]. Notably, plasma levels of liver enzymes showed a time-dependent decrease in those subjects receiving the supplementation compared with placebo [31]. Furthermore, in a trial in patients with diabetes and MASLD, a daily nicotinamide supplementation of 1000 mg daily did not influence liver steatosis or fibrosis [30]. On the other hand, to the best of our knowledge, only one recent cross-sectional study conducted in the United States found that moderate niacin intake was associated with a decrease in the prevalence of MASLD [34].

The present study aimed to assess the association between the intake of niacin and NE and the presence of MASLD in subjects from Catalonia, Northeast Spain. Additionally, because the intake of dietary NE and protein are tightly related, as a secondary objective, we further analyzed whether there was an association between protein intake with the presence of MASLD.

## 2. Materials and Methods

### 2.1. Study Design and Settings

This was a case-control analysis using data from two cohorts previously recruited from Catalonia between March 2010 and July 2014 [35,36,37,38]. The first study included a cohort of individuals with T2DM that aimed to study microvascular disease in patients with T2DM [37,38]. The second study was a population-based cohort designed to determine the prevalence of prediabetes and risk factors associated with this condition [35,36].

### 2.2. Participants

For the purpose of this analysis, we screened all of the subjects from the two cohorts [28,29,30]. We included a total sample of 444 age- and sex-matched subjects: 222 with MASLD and 222 without MASLD. The glucose tolerance status of the study subjects included T2DM, prediabetes, or normal glucose tolerance. The inclusion criteria in the T2DM cohort were age between 40 and 75 and a diagnosis of T2DM. The exclusion criteria were as follows: being a health professional, cognitive impairment (dementia and mental illness), presence of macrovascular complications (heart failure, cerebrovascular disease, ischemic heart disease, and peripheral arterial disease), previous diabetic foot disease, macroalbuminuria (defined as a urine albumin/creatinine ratio greater than 300 mcg/g), renal insufficiency (defined as a glomerular filtration rate < 60 mL/min), and the presence of any other condition requiring specific nutritional therapy (e.g., pregnancy). For the subjects without T2DM, the inclusion criteria were as follows: subjects aged 25 or over attending the Primary Care Centre of the Alt Urgell region of the province of Lleida. Subjects were excluded if they had already been diagnosed with diabetes [type 1 diabetes mellitus (TD1M), T2DM, or other specific subtypes], treatment with oral antidiabetic agents, cardiovascular diseases, history of cancer treated in the last 5 years, non-melanoma skin cancer, renal disease or anemia, hepatitis or other significant liver diseases, gastrointestinal disease, recent abdominal surgery, chronic obstructive pulmonary disease, chronic infectious disease, use glucocorticoids or beta-blockers, and significant psychiatric disorders with psychological symptoms.

Additionally, in this study, we excluded subjects with alcohol consumption exceeding >30 g/day in men and >20 g/day in women [1], individuals with highly unbalanced and/or extreme dietary intake (<1000 kcal/day or ≥4000 kcal/day), high consumption of niacin (mg/day) > 44, NE (mg/day) > 60 and >2.3 g protein /kg weight/day), those lacking anthropometric measurements and biochemical values (gamma-glutamyl transferase, triglycerides) necessary for Fatty Liver Index (FLI) calculation, and individuals without dietary intake data available.

Both cohorts were approved by the Ethics Committee of the University Hospital of Lleida (CEIC 1079) and Ethics Committee of the Primary Health Care University Research Institute (IDIAP) Jordi Gol (PI12/043). Informed consents were obtained from all participants according to the Declaration of Helsinki [35,36,37,38].

### 2.3. Variables

We estimated the presence of MASLD using the FLI formula, which includes the following clinical parameters: triglycerides (mg/dL); body mass index (BMI; kg/m^2^); gamma-glutamyl transferase (GGT; U/L); and waist circumference (WC; cm) [39]. FLI has proven to be a reliable proxy for the diagnosis of hepatic steatosis in current clinical practice and has been proposed as a good predictor of hepatic steatosis in patients diagnosed with MASLD [40,41]. A cut-off point of ≥60 was used in this study to enhance the specificity of the diagnostic tool [42]. FLI ≥ 60, along with one of the three criteria outlined in the new definition consensus for MASLD, was used to define a diagnosis of MASLD, consistent with previous protocols [43]. Variables related to weight, height, blood pressure, BMI, and WC were analyzed [35,37]. Various potential confounding variables were considered a priori, including sociodemographic factors, alcohol intake, current smoking habits, and biochemical measurements.

Physical activity was assessed using the International Physical Activity Questionnaire (IPAQ) validated in Spanish [44], according to the Bernstein et al. [45] method: this was classified as sedentary if less than 10% of daily energy expenditure came from activities requiring at least four METs (metabolic equivalent of tasks, e.g., walking or cycling for over 25 min per day). Regular physical activity was classified if more than 10% of energy expenditure came from such activities. According to the World Health Organization (WHO), one MET is the energy cost of sitting quietly, equivalent to 1 kcal/kg/h [46]. Hypertension and dyslipidemia were considered if patients were on antihypertensive or lipid-lowering medication [36,37]. Prediabetes and diabetes were classified based on the American Diabetes Association (ADA) criteria [47,48].

### 2.4. Dietary Niacin Consumption

Dietary information was collected using the validated Spanish 101-item semi-quantitative food frequency questionnaire (FFQ) administrated by trained researchers face-to-face [49]. The FFQ allows for the measurement of dietary frequency categorized as monthly, weekly, or daily intake of various food groups and collects data on food consumption during the last year prior to the study visit [49,50]. Nutrient intake food consumption data were obtained from US Department of Agriculture composition tables, as well as other food sources and serving sizes in English and Spanish composition tables [51,52,53]. Dietary niacin and NE (mg/day) intakes were obtained from the FFQ. NEs were calculated based on both tryptophan and niacin dietary intake, adjusted for the total energy intake [49]. The adjusted niacin and NE intake were evaluated, taking into account the Recommended Daily Allowance (RDA) intake of niacin and NE according to European Food Safety Authority (EFSA) data. The RDA is 5.5 mg NE1000 kcal per day [13].

### 2.5. Dietary Macronutrient Consumption

All macronutrients were obtained from the FFQ [49]. All these were adjusted for total energy intake (kcal/day). In particular, protein intake was also adjusted for weight using the formula g/kg/day [54]. An established RDA of 0.8 g/kg/day was used as the reference value [54].

### 2.6. Statistical Analysis

Categorical and binary variables were summarized using frequency counts and percentages (n, %), while group differences in numeric variables were assessed using the chi-square test or Fisher’s test. For the comparison of categorical variables with numerical variables, t-student or the Kruskal–Wallis test was used, depending on the distribution of the data. The intake of niacin, NE and protein was stratified into tertiles (T1 = lowest intake, T3 = highest intake), and the association between the presence of MASLD and intake tertiles was analyzed using multivariate logistic regression, with a total of four models (unadjusted model, model 1, model 2, and model 3). Model 1 was adjusted by age and sex, model 2 by age, sex, physical activity level, smoking status, and total caloric intake, and finally, model 3 was adjusted for all the confounding variables (age, sex, sedentary activity, total caloric intake, smoking, BMI, GFR, hypertension, dyslipidemia, and T2DM). Non-linear relationship analysis between the dietary intake of niacin and MASLD was explored using restricted cubic spline (RCS) regression with three knots (10th, 50th, and 90th percentiles). In our study, we selected the number of nodes based on previous studies and statistics guidelines [55]. Specifically, a three-node, non-linear association model was used to warrant an adequate balance between model flexibility and the risk of overfitting, hence allowing an effective interpretability and robustness of the results. A conservative type I error rate of 5% was adopted, with statistical significance set at *p*-value < 0.05. Confidence intervals (CIs) were examined for the presence of the null value (0 for categorical variables and 1 for continuous variables) to ensure the reliability of the findings. The statistical analysis was performed using R statistical software [56].

## 3. Results

Out of the 857 participants from both cohorts, 444 were included after applying the selection criteria. Figure S1 summarizes the flowchart of the study.

Compared with the group of subjects without MASLD (non-MASLD group), subjects with MASLD had lower levels of education (*p* < 0.001), higher weight (*p* < 0.001), WC (*p* < 0.001), and BMI (*p* < 0.001). Additionally, they were more sedentary (*p* = 0.002), had a higher frequency of T2DM (52.7%; *p* < 0.001), dyslipidemia (31.5%; *p* = 0.005), and hypertension (25.4%; *p* < 0.001) (Table 1).

### 3.1. Dietary Intake

Dietary niacin and NE consumption did not differ between study groups (*p*-value = 0.931, *p*-value = 0.800, respectively) (Table 2). Regarding weight-adjusted protein intake, subjects with MASLD had a significantly lower intake (~1.3-fold, *p*-value < 0.001) compared with those without MASLD. There were no differences between the two study groups in the intake of macronutrients (Table 2).

### 3.2. Association Analysis Between Dietary Niacin and MASLD 

**Figure 1:**
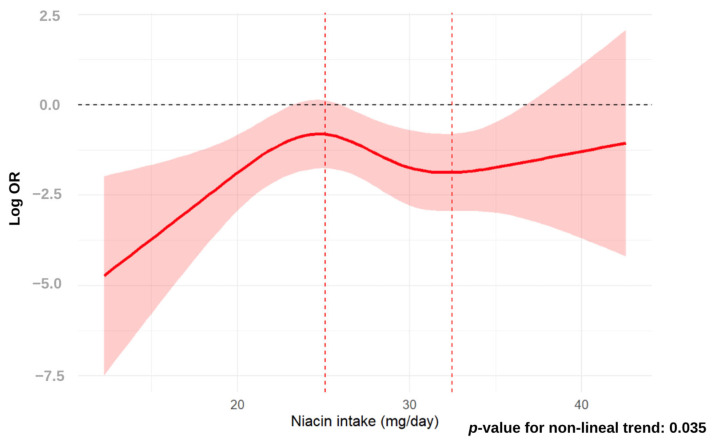
Restricted cubic spline regression analysis of the association between niacin intake (mg/day) and MASLD. RCS regression adjusted for age, sex, total caloric intake, sedentary activity, smoking, GFR, dyslipidemia, hypertension and T2DM. The X-axis represents niacin intake (mg/day) as a continuous variable, and the Y-axis is the Log odds ratio (OR) of the probability of developing MASLD. Greyish red represents the confidence interval (CI). The dashed red lines indicated the inflection points of niacin intake (mg/day).

**Figure 2:**
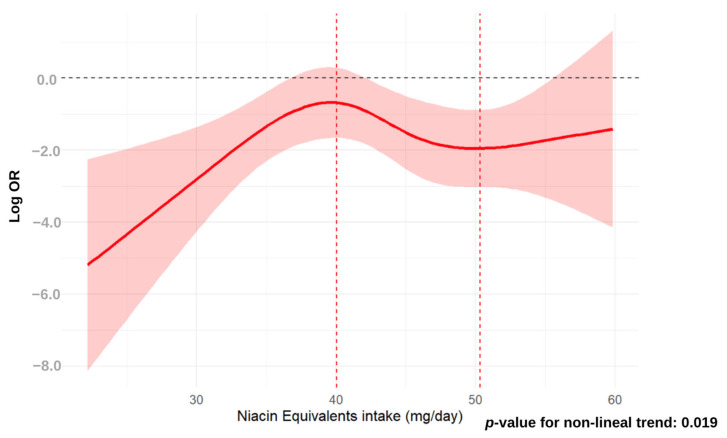
Restricted cubic spline regression analysis of the association between niacin equivalent intake (mg/day) and MASLD. RCS regression adjusted for age, sex, total caloric intake, sedentary activity, smoking, GFR, dyslipidemia, hypertension and T2DM. The X-axis represents the EN intake (mg/day) as a continuous variable, and the Y-axis is the Log ORs (OR) of the probability of developing MASLD. Greyish red represents the confidence interval (CI). The dashed red lines indicated the inflection points of NE intake (mg/day).

Both dietary niacin intake and odds of MASLD were significantly associated (*p*-value = 0.035), as revealed by non-linear dose-response relationship analysis using the RCS approach. The distribution of odds of MASLD across dietary niacin intake showed a maximal peak at a dietary niacin intake of 25 mg/day and a plain valley peak at a dietary niacin intake of 32 g/day. Overall, dietary niacin intake conferred protection when niacin intake values were in the range below 35 mg/day (Figure 1), being significantly related to lower odds of MASLD those niacin intake values below 22 g/day and values within the niacin intake range from >25 to <35 mg/day, respectively. In contrast, for dietary niacin intake values beyond 35 mg/day, the area of CI clearly overlapped the neutral OR line, which was defined by the zero value of the log OR. The RCS shape was similar when the dietary NE intake was considered for non-linear association analysis with MASLD (Figure 2). In this case, the distribution of odds of MASLD across dietary niacin intake showed a maximal peak at a dietary niacin intake of 40 mg/day, and a plain valley at a dietary niacin of 50 g/day. The shape of the curve defined by the odds values across dietary NE intake values was very similar to that built when dietary niacin intake was considered. The range of dietary NE intake values showing lower odds of MASLD was established at intake levels below 55 mg/day, being significantly related to lower odds of MASLD in the range of NE intake values below 35 g/day and from >42.5 to <55 mg/day, respectively. Similarly, for dietary NE intake values beyond >55 mg/day, the area of CI clearly overlapped the neutral OR line defined by the zero value of log OR.

**Figure 3:**
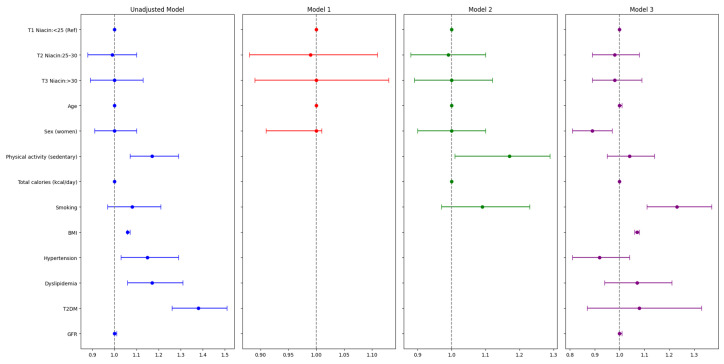
Forest plot of multiple logistic regression of niacin intake (mg/day) in the MASLD group. Data are mean (SD) for continuous variables and number (%) for categorical variables. BMI, body mass index; GFR, glomerular filtration rate; MASLD, metabolic dysfunction-related steatotic liver disease; T2DM, type 2 diabetes. Model 1: adjusted for age and sex. Model 2: adjusted for age and sex, plus physical activity, total calories (kcal/day), and smoking status. Model 3: adjusted for the variables of model 2 plus BMI, hypertension, dyslipidemia, T2DM, and GFR. The blue lines represent the unadjusted model, while the red lines correspond to Model 1, the green lines represent Model 2, and the purple lines correspond to Model 3. The association between MASLD and NE was calculated with the relative effect measure of odds ratios (ORs) and the 95% confidence interval (CI).

**Figure 4:**
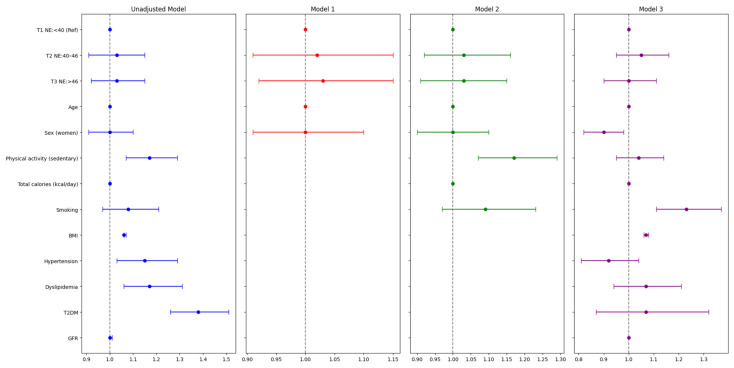
Forest plot of multiple logistic regression of niacin equivalents intake (mg/day) in MASLD group. Data are mean (SD) for continuous variables and number (%) for categorical variables. BMI, body mass index; GFR, glomerular filtration rate; MASLD, metabolic dysfunction-related steatotic liver disease; T2DM, type 2 diabetes. Model 1: adjusted for age and sex. Model 2: adjusted for age and sex, plus physical activity, total calories (kcal/day), and smoking status. Model 3: adjusted for the variables of model 2 plus BMI, hypertension, dyslipidemia, T2DM, and GFR. The blue lines represent the unadjusted model, while the red lines correspond to Model 1, the green lines represent Model 2, and the purple lines correspond to Model 3. The association between MASLD and NE was calculated with the relative effect measure of odds ratios (ORs) and the 95% confidence interval (CI).

Niacin and NE were also considered categorical variables and were analyzed using multivariate logistic regression analysis (Table 3 and Table 4; Figure 3 and Figure 4). Descriptive analysis of both variables showed no statistically significant differences in the proportion of subjects in each tertile between the MASLD and non-MASLD group (Table 3 and Table 4).

The multivariable analysis showed that neither dietary niacin nor NE consumption was associated with MASLD, even after adjusting for confounding variables (Figure 3 and Figure 4; Tables S2 and S3).

Among intrinsic, non-modifiable confounders, the female sex was significantly protective against MASLD, as revealed after adjusting for all relevant confounding variables (Model 3, Figure 3): OR (95% CI) = 0.89 (0.81–0.97) (*p*-value = 0.009). Aging, which is considered another non-modifiable risk factor for MASLD, did not have any statistically significant influence in any of the models.

Regarding other known modifiable risk factors for MASLD, sedentarism failed to remain associated with MASLD in Model 3 (OR (95% CI) = 1.04 (0.95–1.14) *p*-value = 0.366). BMI was associated with MASLD in the fully adjusted model (Model 3, OR (95% CI) = 1.07 (1.06–1.08), *p*-value < 0.001). Smoking was identified as one of the variables positively associated with MASLD (OR (95% CI) = 1.23 (1.11–1.36), *p*-value < 0.001). Finally, other clinical conditions, such as hypertension, dyslipidemia, T2DM, and GFR, were not associated with MASLD (Figure 3; Table S2). Similar results were reached when considering dietary NE as a variable (Figure 2; Table S3).

Protein intake was also analyzed as a categorical variable according to tertiles of intake. The descriptive analysis showed significant differences, with the group without MASLD having a higher protein intake compared with the non-MASLD group (Table S1). In the multivariable analysis, the second and third tertiles of protein intake showed significant protective effects in the fully adjusted model (Model 3, Figure S2; Table S4). Regarding the non-modifiable factors, neither sex nor age were associated with MASLD in any of the analyses. The detrimental effect of smoking was maintained in Model 2 and Model 3 (OR (95% CI) = 1.15 (1.03–1.27), *p* = 0.012; 1.23 (1.11–1.37), *p* < 0.001, respectively). Higher BMI was also associated with a negative effect in Model 3 (OR (95% CI) = 1.07 (1.05–1.08), *p* < 0.001). Total caloric intake, along with MASLD-related comorbidities (hypertension, dyslipidemia, T2DM, and GFR), had no statistically significant effect in Model 3 (Table S4).

## 4. Discussion

Our data showed that both dietary niacin and NE did not differ between subjects with or without MASLD. However, the RCS analysis uncovered a significant non-linear dose-response association between dietary niacin intake and odds of MASLD. Specifically, such analysis revealed that MASLD risk was decreased in subjects with niacin intake values of <35 mg/day. In addition, dietary protein intake (expressed as g/kg/day) was significantly lower in the MASLD group; however, it was not related to incident MASLD. Notably, our analysis also revealed that smoking was consistently associated with MASLD in all the models.

Hepatic steatosis in MASLD is closely linked to reduced liver NAD⁺ levels [18]. Niacin, a precursor of NAD⁺, replenishes these levels, mitigating oxidative stress, inflammation, and lipotoxicity, as shown in preclinical studies [15,16,17,18,19,20,23]. Additionally, niacin may also ameliorate MASLD by, on one side, inhibiting hepatic lipogenesis [29] and, on another, by reducing free fatty acid flux to the liver because of the inhibiting effect on adipose tissue lipolysis via the nicotinic acid-specific receptor GPR109A-mediated signaling pathway [29,33]. The relationship between daily dietary niacin intake and MASLD is poorly studied and elusive. To our knowledge, only two large observational, cross-sectional studies have revealed that higher daily dietary niacin intake is associated with a lower likelihood of MASLD [32,34]. Specifically, an independent study using the NHANES cohort found a U-shaped non-linear dose-response relationship between niacin intake and MASLD [34], with the odds of MASLD showing a gradual reduction along with niacin intake increase until reaching the threshold of 23.6 mg/day, where the odds of MASLD was null, while from this, niacin intake value on the odds MASLD progressively increased along with increasingly higher dietary niacin intake values [34]. In contrast with the latter study, in our study, the range of dietary niacin intakes showing potentially protective odds of MASLD was roughly <35 mg/day. Intriguingly, and similar to us, the logistic regression models did not reveal any association between niacin intake and MASLD, despite adjusting for almost the same confounding variables [34]. In support of this, daily niacin intake was not associated with MASLD in two other independent case-control studies [57,58]. In one of these studies, an Iranian case-control study, daily dietary niacin intake in subjects with MASLD did not differ from that estimated in the control group [58]. Furthermore, niacin intake only showed a marginal, non-significant inverse association with MASLD in another independent Indian case-control study, including 160 cases with MASLD and 160 controls [57]. In both previous studies, the estimated dietary niacin values were much lower than those calculated in our study; in the Indian study, 50% of the participants did not meet the RDA for niacin [57,58].

In general, the daily niacin intake reported in all previous studies was much lower than that calculated for our cohort. In our study, niacin intake ranged from the lowest (less than 25 mg) to the highest (greater than 30 mg); the sources considered to estimate dietary niacin intake included dairy products, eggs, meat, fish, poultry, legumes, and nuts, based on the FFQ with 101 items [49]. For instance, in the three NHANES observational studies, the reported daily niacin intake was much lower than the one calculated in our study [32,34,59]. In the first NHANES study, the lower quartile of daily niacin intake established for the participants of the NHANES cohort study group was <16 g/day (1st quartile) compared with the dietary niacin and NE estimated for our lowest tertile (<25 mg/day; <40 mg/day, respectively) [32]. Similar findings were reported in the other independent NHANES cross-sectional sub-study, where participants were distributed into tertiles according to their daily niacin intakes, in which the lowest tertile included participants displaying values of <18 g niacin/day [59]. Of note, in this last NHANES sub-study, the highest tertile of daily dietary niacin intake (i.e., ≥26.7 mg niacin/day) was not significantly associated with a lower likelihood of MASLD, but the risk of all-cause mortality was lower in participants in the MASLD group with the highest intake of niacin [59]. Finally, in the third study of the NHANES cohort, the mean niacin intake was 22.6 mg/day [34]. The reason(s) for the differences in daily niacin consumption between our study and others is unknown; however, possible explanations could include the use of different methods for dietary data collection, along with different dietary patterns due to, e.g., geographical and ethnic differences in the cohorts [32,57,58,59,60]. Regarding dietary data collection, a study using the NHANES observational data and the Indian case-control study used two 24-hour recall questionnaires to assess niacin intake [32,34,57,59]. In contrast, the Iranian case-control study utilized a valid and reliable 168-item semi-quantitative FFQ [58]. In our study, we used a 101-item semi-quantitative FFQ validated for the Spanish population [49]. Additionally, in previous studies, only niacin intake and not NE was analyzed [32,57,58,59], in contrast to our study, which included both. In general, dietary NE values are frequently higher than dietary niacin values, as NE also includes niacin derived from dietary tryptophan, i.e., from protein breakdown. Consistently, in our cohort, dietary protein intake was not associated with incident MASLD after adjusting for other confounders.

Our study identified a direct relationship between smoking and incident MASLD. This relationship remained significant even after discounting other major confounding variables. Consistent with this observation, a recent meta-analysis revealed a significant association between smoking and MASLD [61]. Although the metabolic basis of this relationship is still under investigation, intake of nicotine has been found to enhance hepatic steatosis in an experimental preclinical model of MASLD [62]. In this regard, the increased release of toxins coming from heavy smoking has been recently reported to induce hepatic necroinflammation and worsening liver lesions to much more active forms, such as fibrosis, in chronic liver disease [63]. Taken together, this body of evidence suggests smoking to be a significant risk factor in the development and progression of liver disease.

Another relevant finding from our study was the contribution of sex to MASLD, whereby females were protected against MASLD across the models. Our observation is aligned with the notion that females are less prone to dysmetabolic features compared with males and with epidemiological data showing that MASLD prevalence and severity are significantly increased in men compared with women [64,65]. Furthermore, consistent with previous studies, our analysis revealed that higher BMI was a risk factor for MASLD [66,67].

One of the main strengths of our study was having data from two cohorts collected during the same period in the Mediterranean areas of Catalonia, enhancing the representativeness of the study sample. Furthermore, to our knowledge, this is the first study to consider NE as a dietary parameter for estimating niacin intake and incident MASLD. This approach is more reliable and realistic as it also considers tryptophan sources [40]. Another strength was the use of the FFQ, which has good reproducibility for estimating dietary intake, with data being collected by trained professionals. However, the current study has several limitations, mainly due to its observational case-control design, which does not allow us to establish causal relationships between the study variables. Additionally, FLI, along with at least one trait of metabolic syndrome, was used as a diagnostic proxy for MASLD, so it is a score calculated using cardiometabolic variables; therefore, data should be interpreted with caution. The maximal levels of dietary niacin estimated from FFQ may not be high enough to exert a favorable effect on MASLD incident; therefore, intervention studies with higher doses of niacin than estimated from the FFQ are warranted to directly assess the favorable influence of niacin on MASLD. Finally, the estimation of dietary niacin can be significantly influenced by the methods used to collect dietary data and information on supplements, making it even more challenging to assess the potential benefits of niacin intake on MASLD in human studies. Further research on dietary niacin intake, particularly through interventional studies, is therefore warranted to develop targeted and specific nutritional recommendations for MASLD management.

## 5. Conclusions

RCS analysis revealed that lower dietary niacin or NE were significantly associated with odds values of MASLD, indicating a negative association with MASLD. However, the fully adjusted logistic regression models did not show any association between dietary intake of niacin or NE and MASLD. Additionally, protein intake was not associated with MASLD after adjusting for the same confounding variables. Instead, our analysis identified the contribution of certain intrinsic characteristics, such as female sex, or modifiable characteristics, such as smoking and BMI, to MASLD. Further clinical studies of interventions with niacin derivatives are warranted to better assess the contribution, if any, to clinical MASLD.

## Figures and Tables

**Table 1:** Clinical and demographic characteristics of study participants.

Characteristics	Non-MASLD (n = 222)	MASLD (n = 222)	*p*-Value
Age, years	55.1 (11.9)	55.2 (11.9)	0.975
Women, n (%)	113 (50.9)	113 (50.9)	1.000
Caucasian, n (%)	218 (98.2)	211 (95.0)	0.096
Secondary high cycle, n (%)	123 (55.4)	95 (42.8)	0.004
Weight, kg	70.2 (10.8)	89.2 (13.3)	<0.001
Height, cm	166 (9.7)	165 (9.3)	0.080
WC, cm	92.1 (9.7)	110 (10.0)	<0.001
BMI, kg/m^2^	25.4 (3.1)	33.0 (5.0)	<0.001
Glucose, mg/dL	102 (30.6)	130 (54.1)	<0.001
HbA1c, %	5.9 (0.9)	6.8 (1.7)	<0.001
sBP, mm/Hg	126 (18.9)	136 (18.4)	<0.001
dBP, mm/Hg	77.0 (9.9)	79.8 (10.9)	0.005
Total cholesterol, mg/dL	203 (34.4)	200 (38.9)	0.360
HDL, mg/dL	59.6 (15.1)	50.5 (12.8)	<0.001
LDL, mg/dL	125 (29.1)	121 (33.1)	0.139
Triglycerides, mg/dL	94.5 (39.9)	163 (134)	<0.001
GGT, U/L	22.6 (30.3)	45.9 (107)	0.002
ALT, U/L	21.3 (25.8)	27.3 (16.6)	0.004
FLI	31.5 (17.1)	81.4 (12.4)	<0.001
GFR	91.0 (13.3)	93.6 (16.2)	0.150
Physically sedentary, n, (%)	76 (34.2)	109 (49.5)	0.002
Smoking, n, (%)	42 (18.9)	54 (24.3)	0.384
Alcohol, g/day	5.25 (6.3)	5.02 (7.1)	0.724
T2DM, n, (%)	50 (22.5)	117 (52.7)	<0.001
Prediabetes, n, (%)	66 (29.7)	48 (21.6)	0.065
Dyslipidemia, n, (%)	43 (19.4)	70 (31.5)	0.005
Hypertension, n, (%)	90 (15.2)	74 (25.4)	<0.001

Data are mean (SD) for continuous variables and number (%) for categorical variables. ALT, alanine aminotransferase; BMI, body mass index; CT, total cholesterol; dBP, diastolic blood pressure; FLI, Fatty Liver Index; GFR, glomerular filtration rate; GGT, gamma-glutamyl transferase; HbA1c, glycosylated hemoglobin; HDL, high-density lipoprotein; LDL, low-density lipoprotein; MASLD, metabolic dysfunction-related steatotic liver disease; sBP: systolic blood; TG, triglyceride; T2DM, type 2 diabetes mellitus; WC, waist circumference.

**Table 2:** Dietary intake of main macronutrients and niacin.

Dietary Intake	Non-MASLD (n = 222)	MASLD (n = 222)	*p*-Value
Energy intake (kcal/day)	2197.0 (533.0)	2168.0 (543.0)	0.569
Carbohydrates (g/day)	219.0 (35.6)	217.0 (36.8)	0.712
Proteins (g/day)	98.4 (14.6)	99.6 (15.8)	0.400
Protein intake (g/kg/day)	1.4 (0.3)	1.1 (0.2)	<0.001
Total fat (g/day)	89.1 (14.4)	89.2 (15.1)	0.923
Saturated fat (g/day)	25.0 (5.7)	24.8 (4.9)	0.805
Monounsaturated fat (g/day)	42.2 (10.1)	42.8 (10.7)	0.533
Polyunsaturated fat (g/day)	15.1 (4.2)	14.9 (4.4)	0.482
Total fiber (g/day)	24.7 (5.5)	24.4 (5.5)	0.517
Fiber soluble (g/day)	3.8 (1.1)	3.9 (1.2)	0.468
Insoluble fiber (g/day)	14.2 (4.6)	14.2 (4.5)	0.915
Niacin (mg/day)	27.2 (5.13)	27.2 (5.16)	0.931
Niacin Equivalents (mg/day)	43.1 (6.9)	43.3 (6.9)	0.800

Data are mean (SD) for continuous variables. All macronutrients and food groups were adjusted for total caloric intake (kcal/day). Protein intake was further adjusted for the body weight (g/kg/day). MASLD, metabolic dysfunction-related steatotic liver disease.

**Table 3:** Niacin consumption (mg/day) in the study groups distributed by tertiles.

	Niacin Intake (mg/day)			
	Tertile 1 (<25) (n = 151)	Tertile 2 (25–30) (n = 157)	Tertile 3 (>30) (n = 136)	*p*-Value
Non-MASLD	75 (49.7)	80 (51.0)	67 (49.3)	0.954
MASLD	76 (50.3)	77 (49.0)	69 (50.7)	

Data are presented as number (%) for the categorical variable niacin intake (mg/day). Niacin intake levels were categorized into tertiles, being: T1 < 25 mg/day, T2 from 25 to 30 mg/day and T3 > 30 mg/day. MASLD, metabolic dysfunction-related steatotic liver disease.

**Table 4:** Dietary niacin equivalents (NE) (mg/day) distributed by tertiles.

	Niacin Equivalents Intake (NE) (mg/day)			
	Tertile 1 (<40) (n = 141)	Tertile 2 (40–46) (n = 154)	Tertile 3 (>46) (n = 149)	*p*-Value
Non-MASLD	73 (51.8)	76 (49.4)	73 (49.0)	0.876
MASLD	68 (48.2)	78 (50.6)	76 (51.0)	

Data are presented as number (%) for the categorical variable NE intake (mg/day). NE intake levels were categorized into tertiles, being: T1 < 40 mg/day, T2 from 40 to 46 mg/day and T3 > 46 mg/day. MASLD, metabolic dysfunction-related steatotic liver disease.

## Data Availability

The data presented in this study are available on request from the corresponding author.

## Supplementary Materials

The following supporting information can be downloaded at https://www.mdpi.com/article/10.3390/nu16234178/s1, Figure S1: Flowchart of inclusion and exclusion criteria of the study; Figure S2: Forest plot of multiple logistic regression of protein intake (g/kg/day) in MASLD group; Table S1: Protein intake (g/kg/day) in the study groups by tertiles categorization; Table S2: Results of multiple logistic regression models for relations between niacin intake and MASLD according to patient sex, age, clinical variables and comorbidities; Table S3: Results of multiple logistic regression models for relations between NE intake and MASLD according to patient sex, age, clinical variables and comorbidities; Table S4: Results of multiple logistic regression models for protein intake (g/kg/day) and MASLD according to patient sex, age, clinical variables and comorbidities.
