# Supplementary material for: Impact of Dietary Niacin on Metabolic Dysfunction-Associated Steatotic Liver Disease in Mediterranean Subjects: A Population-Based Study
Source: Nutrients. 2024 Nov 30;16(23):4178. doi: 10.3390/nu16234178 (PMC11644089; doi:10.3390/nu16234178)
Supplement: Supplementary file 1 [file nutrients-16-04178-s001.zip › nutrients-3312120-supplementary.pdf]

Supplementary material

Impact of dietary niacin on metabolic dysfunction-associated steatotic liver disease in Mediterranean subjects: a population-based study

Maria Antentas†, Marina Idalia Rojo-López†, Pau Vendrell, Minerva Granado-Casas, Estefanía Moreira, Idoia Genua, Berta Fernandez-Camins, Joana Rossell, Julia Niño-Narvión, Alejandra Estefania Moreira, Esmeralda Castelblanco, Emilio Ortega, Bogdan Vlacho, Nuria Alonso, Didac Mauricio, Josep Julve*

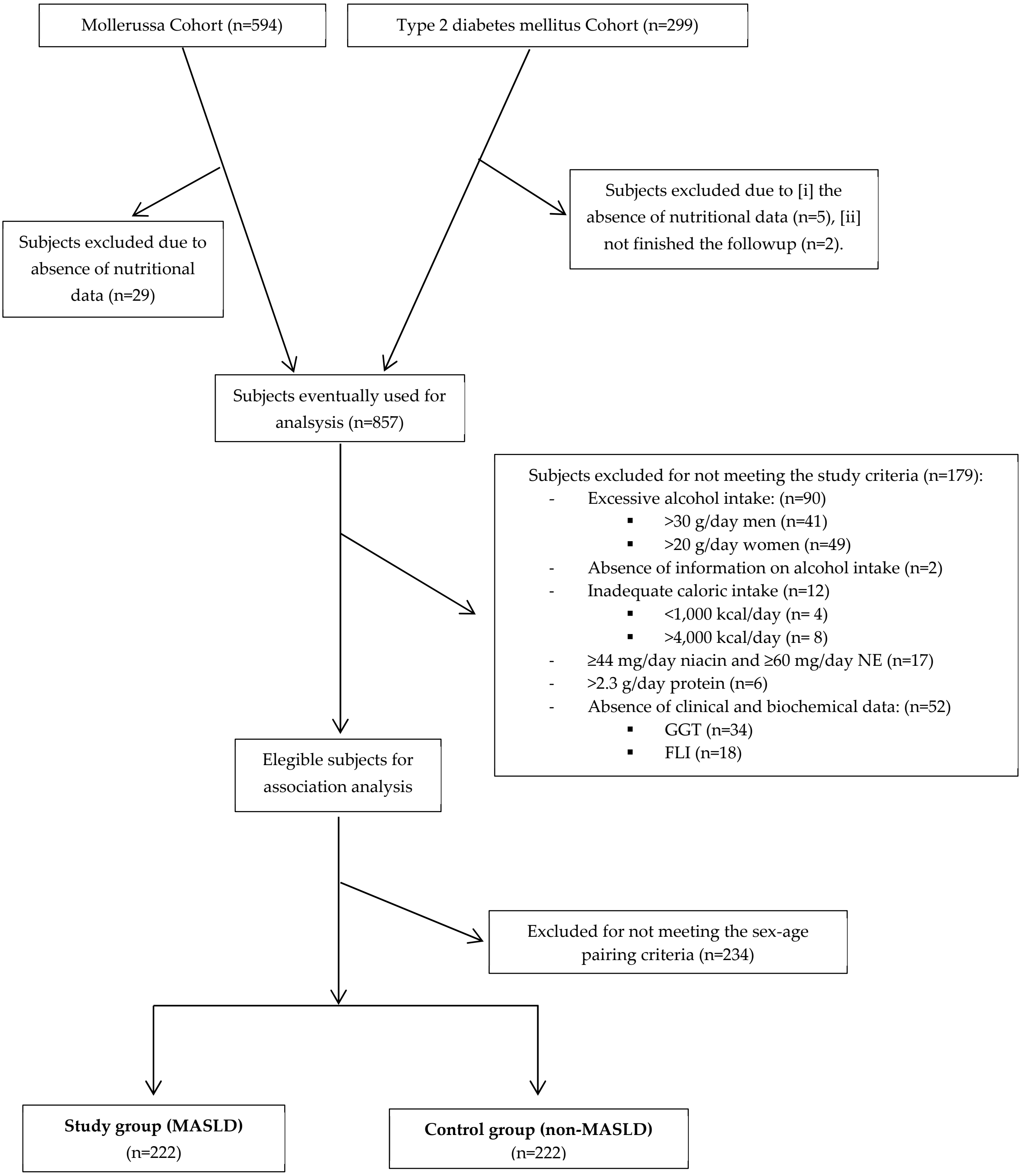

Supplementary Figure S1. Flowchart of the study criteria

Supplemntary Table S1. Protein intake (g/kg/day) in the study groups by tertiles

	Protein Intake (g/kg/day)			
	**Tertile 1 (<1.17) (n=179)**	**Tertile 2 (1.17-1.48) (n=144)**	**Tertile 3 (>1.48) (n=121)**	p-value
**Non-MASLD**	46 (25.7)	80 (55.6)	96 (79.3)	<0.001
**MASLD**	133 (74.3)	64 (44.4)	25 (20.7)	

Data ara presented as number (%) for the categorical variable protein intake (g/kg/day).

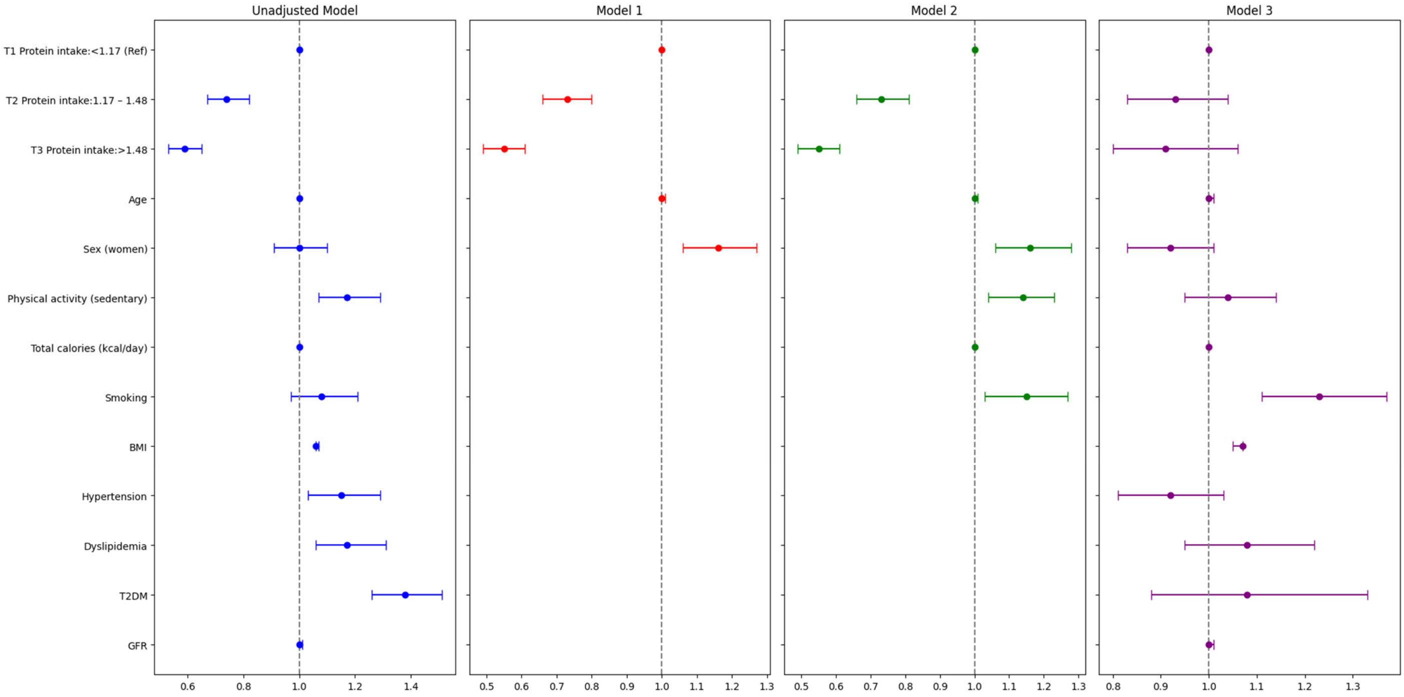

Supplementary Figure SS2. Forest plot of multiple logistic regression of protein intake (g/kg/day) in the MASLD group

BMI, body mass index; GFR, glomerular filtration rate; MASLD, metabolic dysfunction-related steatotic liver disease; T2DM, type 2 diabetes. Model 1: adjusted for age and sex. Model 2: adjusted for age and sex, plus physical activity, total calories (kcal/day) and smoking status. Model 3: adjusted for the variables of model 2 plus BMI, hypertension, dyslipidemia, T2DM and GFR.

The blue lines represent the unadjusted model, while the red lines correspond to Model 1, the green lines represent Model 2, and the purple lines correspond to Model 3.

The association between MASLD and NE has been calculated with the relative effect measure of odds ratios (ORs) and the 95% confidence interval (CI).

Supplementary Table S2. Results of multiple logistic regression models for association between niacin intake and MASLD according to patient sex, age, clinical variables and comorbidities

	MASLD							
	**Unadjusted model**		**Model 1**		**Model 2**		**Model 3**	
**Niacin intake (mg/day)**	OR (CI)	p-value	OR (CI)	p-value	OR (CI)	p-value	OR (CI)	p-value
<25 (Ref)			1.00 (Ref)	1.00 (Ref)	1.00 (Ref)		1.00 (Ref)	
25-30	0.99 (0.88-1.10)	0.822	0.99 (0.88-1.1)	0.823	0.99 (0.88-1.1)	0.808	0.99 (0.89-1.09)	0.703
>30	1.00 (0.89-1.13)	0.946	1.00 (0.89-1.13)	0.946	1.00 (0.89-1.12)	0.970	0.98 (0.89-1.1)	0.758
Age	1.00 (1.00-1.00)	0.975	1.00 (1.00-1.00)	0.977	1.00 (1.00-1.00)	0.857	1.00 (1.00-1.00)	0.605
Sex (women)	1.00 (0.91-1.10)	1.000	1.00 (0.91-1.1)	0.984	0.99 (0.90-1.1)	0.896	0.89 (0.82-0.97)	0.009
Physical activity (sedentary)	1.17 (1.07-1.29)	0.001			1.17 (1.07-1.29)	<0.001	1.04 (0.95-1.14)	0.366
Total calories (kcal/day)	1.00 (1.00-1.00)	0.569			1.00 (1.00-1.00)	0.396	1.00 (1.00-1.00)	0.105
Smoking	1.08 (0.97-1.21)	0.167			1.09 (0.97-1.23)	0.146	1.23 (1.11-1.37)	<0.001
BMI	1.06 (1.06-1.07)	<0.001					1.07 (1.06-1.08)	<0.001
Hypertension	1.15 (1.03-1.29)	0.012					0.92 (0.81-1.04)	0.163
Dyslipidemia	1.17 (1.06-1.31)	0.003					1.07 (0.94-1.21)	0.291
T2DM	1.38 (1.26-1.51)	<0.001					1.08 (0.87-1.33)	0.500
GFR	1.00 (1.00-1.00)	0.133					1.00 (1.00-1.00)	0.119

BMI, body mass index; GFR, glomerular filtratation rate, T2DM, type 2 diabetes. Model 1: adjusted for age and sex. Model 2: adjusted for age and sex, plus physical activity, total calories (kcal/day) and smoking status. Model 3: adjusted for the variables of model 2 plus GFR, BMI, hypertension, dyslipidemia and T2DM. The association between MASLD and niacin has been calculated with the relative effect measure of odds ratios (ORs) and the 95% confidence interval (CI).

Supplementary Table S3. Results of multiple logistic regression models for association between NE intake and MASLD according to patient sex, age, clinical variables and comorbidities

				**MASLD**				
	**Unadjusted model**		**Model 1**		**Model 2**		**Model 3**	
**NE intake (mg/day)**	OR (CI)	p-value	OR (CI)	p-value	OR (CI)	p-value	OR (CI)	p-value
<40 (Ref)			1.00 (Ref)	1.00 (Ref)	1.00 (Ref)		1.00 (Ref)	
40-46	1.03 (0.91-1.15)	0.679	1.02 (0.91-1.15)	0.678	1.03 (0.92-1.16)	0.622	1.05 (0.95-1.16)	0.371
>46	1.03 (0.92-1.15)	0.637	1.03 (0.92-1.15)	0.638	1.03 (0.91-1.15)	0.677	1.00 (0.90-1.11)	0.980
Age	1.00 (1.00-1.00)	0.975	1.00 (1.00-1.00)	0.957	1.00 (1.00-1.00)	0.857	1.00 (1.00-1.00)	0.594
Sex (women)	1.00 (0.91-1.1)	1.000	1.00 (0.91-1.10)	0.998\	1.00 (0.90-1.10)	0.925	0.90 (0.82-0.98)	0.016
Physical activity (sedentary)	1.17 (1.07-1.29)	0.001			1.17 (1.07-1.29)	0.001	1.04 (0.95-1.14)	0.361
Total calories (kcal/day)	1.00 (1.00-1.00)	0.569			1.00 (1.00-1.00)	0.409	1.00 (1.00-1.00)	0.122
Smoking	1.08 (0.97-1.21)	0.167			1.09 (0.97-1.23)	0.153	1.23 (1.11-1.37)	<0.001
BMI	1.06 (1.06-1.07)	<0.001					1.07 (1.06-1.08)	<0.001
Hypertension	1.15 (1.03-1.29)	0.012					0.92 (0.81-1.04)	0.163
Dyslipidemia	1.17 (1.06-1.31)	0.003					1.07 (0.94-1.21)	0.304
T2DM	1.38 (1.26-1.51)	<0.001					1.07 (0.87-1.32)	0.543
FGR	1.00 (1.00-1.01)	0.133					1.00 (1.00-1.00)	0.156

BMI, body mass index; GFR, glomerular filtratation rate, T2DM, type 2 diabetes. Model 1: adjusted for age and sex. Model 2: adjusted for age and sex, plus physical activity, total calories (kcal/day) and smoking status. Model 3: adjusted for the variables of model 2 plus GFR, BMI, hypertension, dyslipidemia and T2DM. The association between MASLD and NE has been calculated with the relative effect measure of odds ratios (ORs) and the 95% confidence interval (CI).

Supplementary Table S4. Results of multiple logistic regression models for protein intake (g/kg weight/day) and MASLD according to patient sex, age, clinical variables and comorbidities

	MASLD							
	**Unadjusted model**		**Model 1**		**Model 2**		**Model 3**	
**Protein intake (g/kg/day)**	OR (CI)	p-value	OR (CI)	p-value	OR (CI)	p-value	OR (CI)	p-value
<1.17 (Ref)			1.00 (Ref)		1.00 (Ref)		1.00 (Ref)	
1.17 – 1.48	0.74 (0.67-0.82)	<0.001	0.73 (0.66, 0.80)	<0.001	0.73 (0.66-0.81)	<0.001	0.93 (0.83-1.04)	0.197
>1.48	0.58 (0.53-0.65)	<0.001	0.55 (0.49, 0.61)	<0.001	0.55 (0.49-0.61)	<0.001	0.92 (0.80-1.06)	0.237
Age	1.00 (1.00-1.00)	0.975	1.00 (1.00, 1.01)	0.372	1.00 (1.00-1.01)	0.188	1.00 (1.00-1.00)	0.502
Sex (women)	1.00 (0.91-1.1)	1.000	1.16 (1.06, 1.27)	0.001	1.16 (1.06-1.28)	0.001	0.92 (0.83-1.01)	0.091
Physical activity (sedentary	1.17 (1.07-1.29)	0.001			1.14 (1.04-1.24)	0.003	1.04 (0.95-1.14)	0.370
Total calories (kcal/day)	1.00 (1.00-1.00)	0.569			1.00 (1.00-1.00)	0.529	1.00 (1.00-1.00)	0.097
Smoking	1.08 (0.97-1.21)	0.167			1.15 (1.03-1.27)	0.012	1.23 (1.11-1.37)	<0.001
BMI	1.06 (1.06-1.07)	<0.001					1.07 (1.05-1.08)	<0.001
Hypertension	1.15 (1.03-1.29)	0.012					0.92 (0.81-1.03)	0.155
Dyslipidemia	1.17 (1.06-1.31)	0.003					1.07 (0.95-1.22)	0.251
T2DM	1.38 (1.26-1.51)	<0.001					1.08 (0.88-1.33)	0.477
GFR	1.00 (1.00-1.00)	0.133					1.00 (1.00-1.00)	0.109

BMI, body mass index; GFR, glomerular filtratration rate, T2DM, type 2 diabetes. Model 1: adjusted for age and sex. Model 2: adjusted for age and sex, plus physical activity, total calories (kcal/day) and smoking status. Model 3: adjusted for the variables of model 2 plus GFR, BMI, hypertension, dyslipidemia and T2DM. The association between MASLD and protein has been calculated with the relative effect measure of odds ratios (ORs) and the 95% confidence interval (CI).
